# The potential function and clinical application of FGF21 in metabolic diseases

**DOI:** 10.3389/fphar.2022.1089214

**Published:** 2022-12-21

**Authors:** Zhiwei Chen, Lili Yang, Yang Liu, Ping Huang, Haiyan Song, Peiyong Zheng

**Affiliations:** ^1^ Institute of Digestive Diseases, Longhua Hospital, Shanghai University of Traditional Chinese Medicine, Shanghai, China; ^2^ Teaching Experiment Center, Shanghai University of Traditional Chinese Medicine, Shanghai, China

**Keywords:** fibroblast growth factor 21, metabolic dysfunction, obesity, type 2 diabetes mellitus, non alcoholic fatty liver disease

## Abstract

As an endocrine hormone, fibroblast growth factor 21 (FGF21) plays a crucial role in regulating lipid, glucose, and energy metabolism. Endogenous FGF21 is generated by multiple cell types but acts on restricted effector tissues, including the brain, adipose tissue, liver, heart, and skeletal muscle. Intervention with FGF21 in rodents or non-human primates has shown significant pharmacological effects on a range of metabolic dysfunctions, including weight loss and improvement of hyperglycemia, hyperlipidemia, insulin resistance, cardiovascular disease, and non-alcoholic fatty liver disease (NAFLD). Due to the poor pharmacokinetic and biophysical characteristics of native FGF21, long-acting FGF21 analogs and FGF21 receptor agonists have been developed for the treatment of metabolic dysfunction. Clinical trials of several FGF21-based drugs have been performed and shown good safety, tolerance, and efficacy. Here we review the actions of FGF21 and summarize the associated clinical trials in obesity, type 2 diabetes mellitus (T2DM), and NAFLD, to help understand and promote the development of efficient treatment for metabolic diseases *via* targeting FGF21.

## 1 Introduction of FGF21

So far, there are 23 fibroblast growth factors (FGFs) have been isolated (Szczepańska and Gietka-Czernel, 2022). Classically, FGFs exert the role of regulating cell growth and differentiation, embryonic development, tissue injury repair, angiogenesis, *etc.* (Beenken and Mohammadi, 2009; Chen et al., 2022). They are divided into seven subfamilies, which exert their functions through different modes including autocrine/paracrine, intracrine, and endocrine (Itoh and Ornitz, 2011). The FGF endocrine subfamily includes three members: FGF19 (FGF15 in mice), FGF21, and FGF23 (Kurosu et al., 2007). Unlike other FGFs, these members have weak or no heparin-binding affinity, which renders them into the blood circulation and action as an endocrine hormone (Zhang et al., 2006; Goetz et al., 2007).

The FGF21 genes were originally cloned *via* a PCR approach by Nobuyuki Itoh’s group (Kharitonenkov and Adams, 2014). It shares about 75% of the amino acid sequence between mouse and human (Nishimura et al., 2000). The gene was first defined as a new FGF expressed in the liver (Flippo and Potthoff, 2021). Further studies find that, under physiological conditions, although FGF21 mRNA can be detected expressed in other tissues, such as adipose tissues, pancreas, and muscle (Fon Tacer et al., 2010; Bondurant et al., 2017), circulating FGF21 is still primarily derived from the liver in both rodents and humans (Markan et al., 2014; Hansen et al., 2015). But notably, the nature of the stimulus determines the tissue origins of FGF21. For example, FGF21 expression in liver tissues could be induced by fasting and a ketogenic diet (Badman et al., 2007; Inagaki et al., 2007), whereas overfeeding and obesity-inducing factors in the pancreas and white adipose tissue (WAT) (Oishi et al., 2011; Singhal et al., 2016a; Lundsgaard et al., 2017), by cold exposure in brown adipose tissue (BAT) (Chartoumpekis et al., 2011; Hondares et al., 2011; Keipert et al., 2015), and by exercises in skeletal muscle (Tanimura et al., 2016). Thus, FGF21 is regarded as a stress-induced hormone, which elevates due to metabolism-associated state changes.

To act on the target tissues, FGF21 needs to bind to the FGF receptors (FGFRs) (predominantly FGFR1c) and the obligatory coreceptor protein β-klotho (KLB) (Spann et al., 2021). Neither KLB nor FGFRs can be activated by FGF21 alone (Hayden et al., 2006). KLB is considered to function as a targeting receptor of FGF21, thus promoting binding with the effector receptor FGFR1c (Lee et al., 2018). The formation of the heterodimeric FGFR1c/KLB complex subsequently activates the intracellular tyrosine kinase domains of FGFR1c *via* phosphorylation by ERK and passes on downstream signaling (Kharitonenkov et al., 2005; Kurosu et al., 2007; Ogawa et al., 2007; Yie et al., 2012). However, ERK1/2 activation cannot mediate all of the complicated FGF21 actions. By far, the precise downstream molecular signaling pathways of FGF21 to mediate its multiple functions remain unclarified. FGFR1c is expressed by ubiquitous tissues, whereas KLB expression is restricted to several specific metabolic tissues such as the pancreas, liver, and adipose tissue (Fon Tacer et al., 2010), and lower expression could be detected in the brain (Jensen-Cody et al., 2020). Therefore, the KLB location somehow confers specificity for FGF21 signaling.

## 2 FGF21 actions on target tissues

**FIGURE 1:**
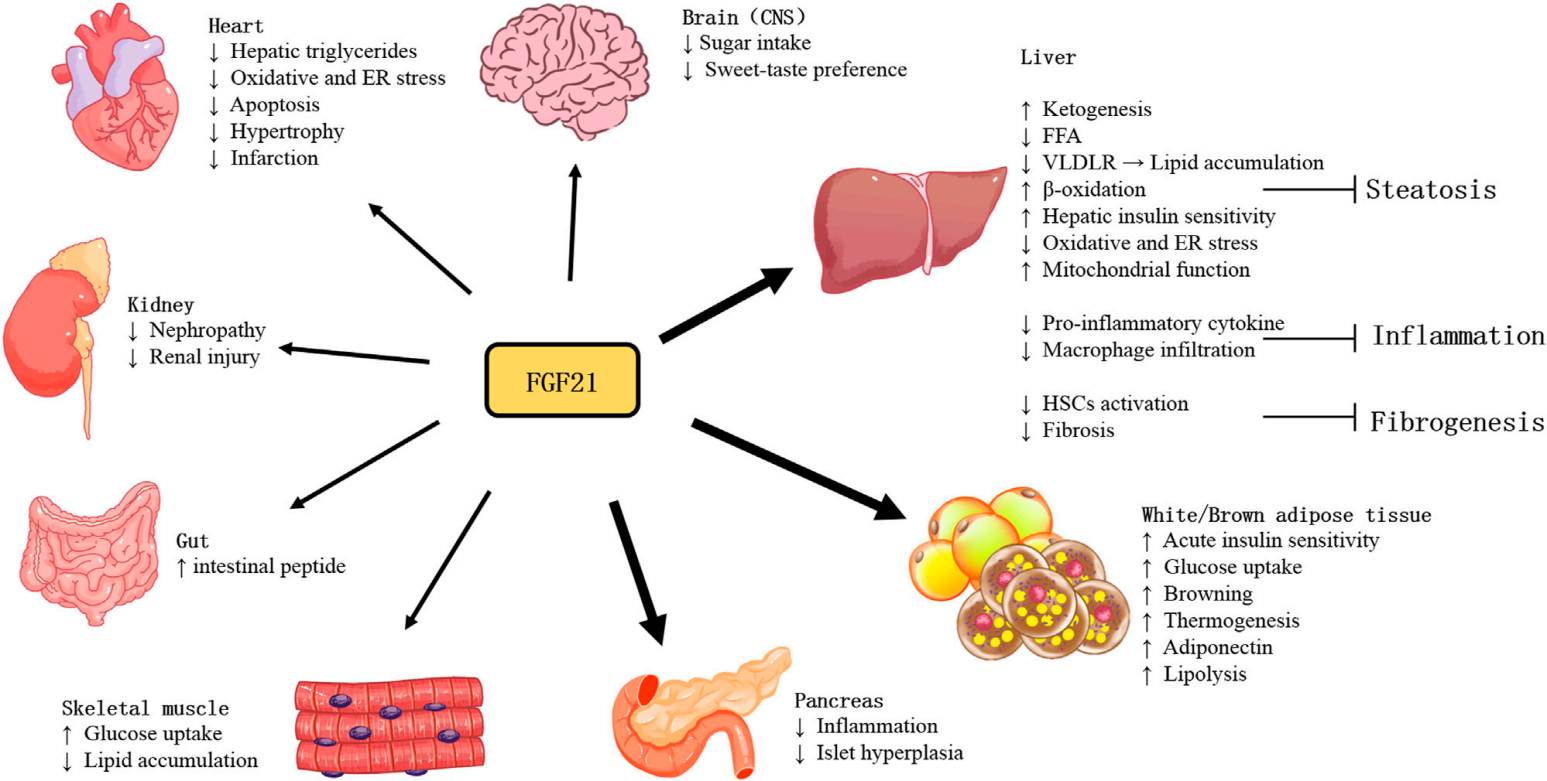
Tissue-specific actions of FGF21.

With more studies of FGF21 performed in recent years, the actions and pathophysiology of this stress-inducible hormone have been revealed. As shown in Figure 1, owing to various tissues of expression and actions, the function of FGF21 is quite complicated. Furthermore, as an endocrine hormone, FGF21 regulates nutrient metabolism and energy homeostasis *via* mediating multi-organ communications.

### 2.1 Central nervous system (CNS)

FGF21 has been demonstrated expressed in brain regions, including the substantia nigra, striatum, hippocampus, and cortex (Makela et al., 2014), it is produced by glial cells, as evidenced by immunoblotting results (Makela et al., 2014). Besides being produced in the brain, blood FGF21 can also cross the blood-brain barrier by simple diffusion and can be detected in human cerebrospinal fluid and mouse brains (Hsuchou et al., 2007). KLB is also expressed in several brain areas, including the suprachiasmatic nucleus (SCN); the dorsal vagal complex (DVC) of the hindbrain, in the area postrema (AP); the nucleus tractus solitarii (NTS); the nodose ganglia (Bookout et al., 2013) and the paraventricular nucleus (PVN) (Liang et al., 2014). Moreover, FGFRs are broadly expressed in CNS (Fon Tacer et al., 2010). FGFR1c is also predominantly expressed in the brain and associated areas (Fon Tacer et al., 2010). The expression of FGF21 in the brain and the presence of its receptor and coreceptor suggest that FGF21 has a potential regulatory role in CNS.

The protein restriction could induce changes in macronutrient preference, energy expenditure, and metabolism (Cummings et al., 2018). FGF21 was significantly increased by protein restriction (Muller and Tschop, 2014). During protein restriction, FGF21 signaling is a pivotal messenger of physiological changes in metabolism and nutrient preferences in the brain. The mice without FGF21 signaling in the brain cannot mediate adaptive metabolic responses to protein restriction and make changes in food preference (Hill et al., 2019). Liver response to high carbohydrates also induces FGF21 expression (Fisher et al., 2017; Lundsgaard et al., 2017). FGF21 enters the circulation to act on the CNS to inhibit simple sugar intake and sweet-taste preference in mice and monkeys (Talukdar et al., 2016a; von Holstein-Rathlou et al., 2016). Notably, FGF21 appears to consume only carbohydrates without reducing total caloric intake in mice (von Holstein-Rathlou et al., 2016). In one clinical study, 7 weeks of daily consumption of sugar-sweetened beverages led to elevated fasting FGF21 in healthy men, irrespective of the sugar type. And it is proposed that sweet-taste food may contribute to the increased FGF21 observed in subjects with metabolic syndrome that is possibly associated with decreased FGF21 response (FGF21 resistance) (Geidl-Flueck et al., 2022). These studies suggest the function of FGF21 in regulating the intake of nutrients.

Recent studies have shown that FGF21 regulates monosaccharide intake and sweet taste preference through signaling to glutamatergic neurons directly (Jensen-Cody et al., 2020). Physiologically, FGF21 signaling to neurons in the paraventricular nucleus regulates basal sucrose intake, whereas FGF21-mediated inhibition of simple-sugar intake requires signaling to neurons in the ventromedial hypothalamus (VNH) (Jensen-Cody et al., 2020). In response to elevated blood glucose concentrations, FGF21 achieves specificity to inhibit monosaccharide intake by enhancing the excitability of KLB^+^ glucose-responsive neurons in the VMH (Jensen-Cody et al., 2020; Flippo and Potthoff, 2021).

### 2.2 Adipose tissues

There are three types of adipocytes: white, beige, and brown adipocytes. White adipocytes are known for storing energy. Brown adipocytes consume energy to produce heat through adaptive thermogenesis (Rosen and Spiegelman, 2014), which requires the gene mitochondrial uncoupling protein 1 (UCP1) (Cannon and Nedergaard, 2004). Cold exposure or β-adrenergic signaling could induce PGC-1α expression, which is a coactivator to drive the expression of UCP1 (Barbera et al., 2001). BAT depots in rodents (Bartelt et al., 2011) and humans increase glucose uptake in response to activation and are highly insulin sensitive (Orava et al., 2011). FGF21 could promote the thermogenic activity of BAT and the browning of WAT (Fisher et al., 2012). Mounting studies have found the relative involvement of FGF21-signaling in the thermogenic response to cold and diet. Mice lacking FGF21 showed an impaired ability to adapt to chronic cold exposure and reduced browning of WAT (Fisher et al., 2012). Adipose-derived FGF21 upregulated expression of UCP1and other thermogenic genes in adipose tissue in an autocrine/paracrine manner (Fisher et al., 2012). FGF21 regulates this process by increasing PGC-1α protein expression in adipose tissues at least in part (Fisher et al., 2012). Moreover, UCP1 is required for FGF21-mediated enhancement of energy expenditure and glucose tolerance *in vivo* (Kwon et al., 2015; Samms et al., 2015). The rapid glucose clearance by FGF21 is defective in the absence of UCP1-dependent thermogenesis, which could increase glucose disposal (Keipert et al., 2020).

FGF21 can promote the uptake of glucose (Kharitonenkov et al., 2005) and lipid (Schlein et al., 2016), and lipogenesis in adipose tissues (Dutchak et al., 2012), thereby protecting the liver and skeletal muscle tissues against ectopic lipids accumulation. In WATs, FGF21 stimulates glucose entry through AMP-activated protein kinase (AMPK) -SIRT1-PGC-1α signaling dependent mechanism, regulates lipolysis, increases mitochondrial oxidative capacity, and enhances the effect of PPAR-γ (Schreuder et al., 2010).

The most abundant adipokine adiponectin was considered the important mediator for FGF21 function. FGF21 administration markedly increased the expression and secretion of adiponectin in adipocytes in mice. In adiponectin-deficient mice, the effects of FGF21 including alleviation of obesity-associated insulin resistance, hyperglycemia, hyperlipidemia, and liver steatosis were reduced. However, circulating FGF21 levels are increased whereas plasma adiponectin concentrations are reduced in both animals and humans with obesity. This might be due to FGF21 resistance, and also suggests that the dysfunctional FGF21-adiponectin axis contributes to the pathogenesis of obesity-related metabolic syndrome (Lin et al., 2013). Long-term HFD-induced obese mice with metabolic dysfunction of glucose and lipid, accompanied by reduced expression of FGFR1 and KLB in adipose tissues, which were markedly reversed by treadmill exercise. Exercise could protect against HFD-induced decreased ability of FGF21 to promote adiponectin secretion, which may be due to the increased expression of FGFR and KLB (Geng et al., 2019; Yang et al., 2019).

FGF21 can significantly improve carbohydrate and lipid homeostasis and promotes weight loss in animal models of obesity and diabetes (Markan and Potthoff, 2016). Studies have shown that FGF21 has both acute and chronic effects on the regulation of metabolism. A single dose of FGF21 administered to obese mice could rapidly increase insulin sensitivity and reduce blood glucose levels by more than 50% (Xu et al., 2009). Using mice with KLB specifically deficient in adipose tissues, it has been found that FGF21 requires direct signaling to brown adipocytes to exert its acute insulin-sensitizing effects (Bondurant et al., 2017). In contrast, long-term administration of FGF21 increased energy expenditure and resulted in weight loss in animal models (Coskun et al., 2008; Xu et al., 2009), and therefore increases insulin sensitivity indirectly, which was independent of FGF21 signaling to adipose tissue and adiponectin production (Bondurant et al., 2017).

### 2.3 Liver

As the major FGF21-producing organ, the liver could also provide an autocrine source of FGF21. Circulating FGF21 is mainly of hepatic origin (Markan et al., 2014). Hepatic FGF21 expression is primarily regulated by nutritional stress, especially starvation (Fazeli et al., 2015). FGF21 expression in the liver is regulated by the peroxisome proliferator-activated receptor-α (PPARα) pathway and cyclic adenosine monophosphate (EPAC)/protein kinase A (PKA) pathway (Lundasen et al., 2007; Cyphert et al., 2014). The PPARα pathway is activated by FFAs and/or protein insufficiency, which increases FGF21 gene expression (Lundasen et al., 2007). Activated by a signaling cascade with the stimulation of the hepatic glucagon receptor, the EPAC/PKA signaling pathway increases FGF21 gene expression and secretion through pre- and post-transcriptional mechanisms (Cyphert et al., 2014).

The c-Jun NH2-terminal kinase (JNK) signaling pathway is stress-responsive and could be activated by nutritional stress, including HFD consumption. The hepatic JNK2α activation can downregulate FGF21 expression by suppressing PPARα and thereby result in systemic metabolism changes. As a heterodimeric partner of PPARα, the JNK substrate retinoid X receptor α (RXRα) phosphorylation at site Ser260 has been found required for suppression of FGF21 expression (Vernia et al., 2022).

Ketogenic diets are employed to challenge metabolic pathways. This kind of diet limits carbohydrate intake and converts the main energy source to ketones, the products of fatty acid metabolism. Typically, mice fed the ketogenic diet lost weight and had elevated circulating FGF21 levels. In contrast, FGF21 null mice gained weight and developed obvious hepatic steatosis after ketogenic diet ingestion (Badman et al., 2009). This aberrant response to the ketogenic diet was associated with reduced levels of β-hydroxybutyrate, suggesting the requirement of FGF21 for fatty acid oxidation (Badman et al., 2009). This effect was evidenced by a significant increase in liver β-oxidation in FGF21^+/+^ mice (Potthoff et al., 2009) and impaired ketone production in FGF21deficient mice (Badman et al., 2009). Indeed, PPARα induces FGF21, which promotes lipolysis in WAT and liver through endocrine and autocrine/paracrine mechanisms, stimulating ketogenesis *in vivo* (Inagaki et al., 2007).

The regulating function of glucose and lipid metabolism of FGF21 is of primary importance in improving liver fibrosis. FGF21 has shown anti-inflammatory and anti-fibrotic effects in the liver. Feeding a methionine-choline deficient diet (MCD) results in lipotoxicity and is associated with a significant increase in FGF21 expression at hepatic and circulating levels. Lipotoxicity is significantly aggravated when lacking FGF21 (Fisher et al., 2014; Tanaka et al., 2015). FGF21-deficient mice showed an increased inflammatory response, increased hepatic macrophage infiltration, and increased expression of pro-inflammatory and pro-fibrotic cytokines (Liu et al., 2016). Conversely, upregulating FGF21 expression in mice by adeno-associated viral vector-mediated gene therapy inhibited hepatic macrophage infiltration (Jimenez et al., 2018), and pharmacological doses of FGF21 suppressed the pro-inflammatory cytokine levels in the liver (Wang et al., 2018; Yin et al., 2018). Preclinical studies have demonstrated that FGF21 has an anti-inflammatory effect and that FGF21 can reduce hepatic immune cell infiltration in mice (Bao et al., 2018). It has been suggested that the mechanism underlying its anti-inflammatory effect may be the down-regulation of IL17A production through the FGF21-adiponectin IL17A axis (Bao et al., 2018). Studies evidenced that exogenous FGF21 can reduce the liver fibrosis degree in the metabolic model of mice (Coskun et al., 2008; Lee et al., 2016). FGF21 treatment downregulated fibrosis markers alpha-smooth muscle actin (α-SMA) and collagen I (Yao et al., 2012) in the liver tissues of MCD diet-induced NASH mice (Le CT et al., 2018).

Increasing evidence demonstrates that bile acids play an insulin-sensitizing role through interaction with its receptor farnesoid X receptor (FXR) (Monte et al., 2009; Mudaliar et al., 2013). However, because its toxic hydrophobic chemicals could damage cell membranes (Attili et al., 1986), bile acids can induce inflammation, fibrosis, and necrosis of the cells, leading to many liver and bile duct diseases (Zakharia et al., 2018). Studies have shown that bile acids and FXR agonists increased the expression and secretion of FGF21 (Cyphert et al., 2012). In hepatocytes and different animal models, FGF21 acutely induced ERK phosphorylation and inhibited Cyp7A1 mRNA expression, significantly reducing bile acid levels in the liver and small intestine (Chen et al., 2018). In obese cynomolgus monkey models, long-term administration of FGF21 analogs inhibited plasma levels of total bile acid and 7α-hydroxy-4-cholesten-3-one (Chen et al., 2018), a biomarker for bile acid synthesis, suggesting the important role of FGF21 in regulating bile acid metabolism as a negative regulator of bile acid synthesis.

Under normal physiological conditions, FGF21 reduces oxidative stress by upregulating Nrf2-mediated antioxidant capacity (Yu et al., 2015). In mice, the administration of exogenous FGF21 improves mitochondrial function in hepatocytes (Chau et al., 2010; Lee et al., 2016), whereas FGF21 deficiency increases hepatic ROS accumulation, which can be alleviated by FGF21 supplementation (Ye et al., 2014). Endoplasmic reticulum (ER) stress is involved in promoting hepatic steatosis, inflammation, and apoptosis. FGF21 is reported to attenuate the process to mitigate NASH development. FGF21 is induced in response to ER stress, which is considered a compensatory mechanism to attenuate ER stress-induced liver lipotoxic injury (Kim et al., 2014). Furthermore, FGF21 may protect against hepatic steatosis by attenuating ER stress-induced VLDL receptor (VLDLR) upregulation and suppressing the maturation level of SREBP1 protein induced by ER stress (Zarei et al., 2018)^,^ (Jiang et al., 2014).

However, in the liver, the predominant expression of FGFR is FGFR4, which has a low affinity for FGF21, whereas the major FGF21 receptor FGFR1 has only a low expression. Therefore, although hepatic FGF21 can influence liver physiology, several studies have suggested that autocrine FGF21 may not be a master regulator of hepatic energy homeostasis. Its profound effects on lipid and glucose metabolism may be through indirect mechanisms (Yang et al., 2012; Li, 2019).

### 2.4 Heart

Initial studies suggested that the FGF21 coreceptor KLB was underexpressed in the heart, which was thus not a primary target of FGF21 (Fon Tacer et al., 2010). However, subsequent studies revealed that relatively stable levels of FGFR1 in addition to KLB were expressed in the heart, which is also an organ source of FGF21 (Tezze et al., 2019).

Exogenous FGF21 could attenuate oxidative stress in cultured cardiomyocytes *in vitro* (Johnson et al., 2009) and prevent cardiac hypertrophy and myocardial infarction in mice (Joki et al., 2015). Mice lacking FGF21 showed increased rates of cardiac hypertrophy and inflammation and decreased capacity of fat oxidation (Planavila et al., 2013). FGF21 can enhance antioxidant activity, thereby inhibiting oxidative stress and endoplasmic reticulum stress (ERS) (Zhang et al., 2021). In atherosclerotic mice, FGF21 treatment markedly reduced lipid deposition and plaque area in the aortic root and reduced lesion severity (Liu et al., 2022). Mover, FGF21 can further reduce endothelial cell injury and apoptosis, thus inhibiting the development of atherosclerosis (Planavila et al., 2015). In animal models, after myocardial ischemia, plasma and cardiac FGF21 levels significantly increased quickly 1 h after coronary artery ligation and continued to increase after 25 h and 1 week (Patel et al., 2014) (Sunaga et al., 2019). In addition, liver-specific deficiency of FGF21 in mice with myocardial infarction resulted in further worsening of cardiac dysfunction (Tang et al., 2018). Preclinical Studies *in vivo* demonstrated the involvement of FGF21 in the pathophysiologic mechanism of heart failure *via* protection against cardiac hypertrophy, oxidative stress, and inflammation in cardiomyocytes. However, the clinical literature showed FGF21 levels paradoxically raised or unchanged in HF and coronary artery disease (Tucker et al., 2022).

### 2.5 Pancreas

The FGF21 expression level in pancreatic acinar tissue is 20 times higher than that in islets, and the pancreatic FGF21 is nutritionally regulated (Singhal et al., 2016b). But both acinar and islet cells are the targets of FGF21. The administration of FGF21 leads to phosphorylation of the downstream ERK1/2 in approximately half of the acinar cells and a small fraction of islet cells (Singhal et al., 2016a).

Although FGF21 expression is high in the pancreas, little is known about the function of FGF21 in this tissue. Studies suggest a regulatory role of FGF21 in the tissue injury induced by experimental pancreatitis, in which FGF21 null mice produced more severe damage than wild-type mice, whereas mice with overexpressing FGF21 showed an attenuated phenotype (Johnson et al., 2009; Johnson et al., 2014). Further study revealed that the transcription factor MIST1 was an upstream regulator for FGF21, and MIST1 deletion resulted in significantly reduced pancreatic FGF21 levels through epigenetic silencing, thereby increasing susceptibility to pancreatitis (Johnson et al., 2014). In a streptozotocin-induced diabetes model, FGF21 was found to play a role in enhancing islet transplantation survival (Uonaga et al., 2010). Moreover, FGF21 also promoted β-cell survival and protects isolated rat islets and insulin-producing INS cells from glucolipotoxicity and cytokine-induced apoptosis (Wente et al., 2006). The effect of FGF21 on insulin or glucagon secretion in islets isolated from healthy animals has not been reported (Xu et al., 2009). In contrast, FGF21 stimulated insulin secretion in the islets isolated from diabetic animals (Wente et al., 2006). But islets from the obese diabetic db/db mice failed to respond to FGF21, possibly as a result of reduced KLB expression (So et al., 2013). The role of FGF21 in the pancreas was further supported by the results of 16-week HFD-fed FGF21-null mice, which developed severe islet hyperplasia and inflammatory infiltration in the periductal region (Singhal et al., 2016b).

### 2.6 Skeletal muscle

Skeletal muscle is the important tissue for systemic insulin-mediated glucose uptake. Recently, skeletal muscles have been found as an important source of FGF21 in both mice and humans during physiological or pathological conditions, including exercise and mitochondrial dysfunction (Tezze et al., 2019). Thus, FGF21 is also defined as a kind of myokine. Generally, it is not considered a target tissue for FGF21 action owing to the lack of KLB expression (Ito et al., 2000; Suzuki et al., 2008). However, previous studies have observed that FGF21 played a direct role in enhancing glucose uptake in skeletal muscle by a mechanism mediated by GLUT1/4 and dependent on atypical PKC-ζ (Mashili et al., 2011; Rosales-Soto et al., 2020). In addition, FGF21 can improve insulin signaling downstream of mouse skeletal muscle by inhibiting mTORC1, subsequently inhibiting the phosphorylation of IRS1 at Ser636/639 and improving insulin sensitivity (Lee et al., 2012). Moreover, long-term administration of FGF21 significantly reduced intramuscular triglyceride levels in an HFD-induced obese mouse model, while these effects disappeared in adiponectin-knockout mice (Lin et al., 2013). This suggests that FGF21 may play a role in skeletal muscle in adiponectin - dependent manner. In summary, FGF21 plays an important role in glucose homeostasis, insulin sensitivity, and lipid metabolism in skeletal muscle either directly or indirectly.

### 2.7 Kidney

FGFRs are also found expressed and localized in adult and developing murine kidneys (Cancilla et al., 1999; Cancilla et al., 2001). Interestingly, FGF21 and its receptors were significantly upregulated in db/db mouse kidneys, which was considered to indicate FGF21 resistance. Exogenous FGF21 treatment significantly down-regulated FGF21 receptor components and activated ERK phosphorylation (Kim et al., 2013). In type 2 diabetic nephropathy, FGF21 significantly reduced urinary albumin excretion and mesangial expansion, and inhibit fibrillary molecular synthesis. On the other hand, FGF21 improved lipid metabolism and oxidative stress injury of kidneys. Thus, FGF21 protected against renal injury through the improvement of insulin resistance, systemic metabolic disorder, and antifibrotic effect (Kim et al., 2013).

### 2.8 Gut

In lactating dams, lactation could induce the hepatic production of FGF21, which is then transferred from plasma to milk and reaches the neonatal intestine. FGF21 activates the FGFR-KLB complex in neonatal intestinal epithelial cells, promoting the production of intestinal peptides involved in the regulation of intestinal function (Gavalda-Navarro et al., 2015).

## 3 The development of FGF21-based drugs for metabolic diseases

Due to the overnutrition and sedentary modern lifestyle, the global prevalence of metabolic diseases such as obesity, T2DM, and NAFLD in the world remains increasing, which are mutually affected risk factors. For example, non-alcoholic steatohepatitis (NASH), the severe stage of NAFLD may involve about 1.5%–6.5% of the general population and as many as 37% of people with type 2 diabetes (T2D) (Younossi et al., 2019). But so far, there is still a lack of specific drugs for these diseases. As accumulating evidence has demonstrated the important roles of FGF21 in regulating glucose and lipid homeostasis through multiple aspects and inter-organ crosstalk, FGF21 is considered a potential target for metabolic abnormalities.

In the clinical study, the aberrant FGF21 expression has been found in different diseases. Through a genome-wide association study (GWAS), circulating FGF21 has been identified as possessing a strong causal effect on improved hyperlipidemia and liver function biomarkers including fibrosis (Larsson et al., 2022). And it was reported that elevated circulating levels of FGF21 were associated with cardiovascular disease (CVD).

**FIGURE 2:**
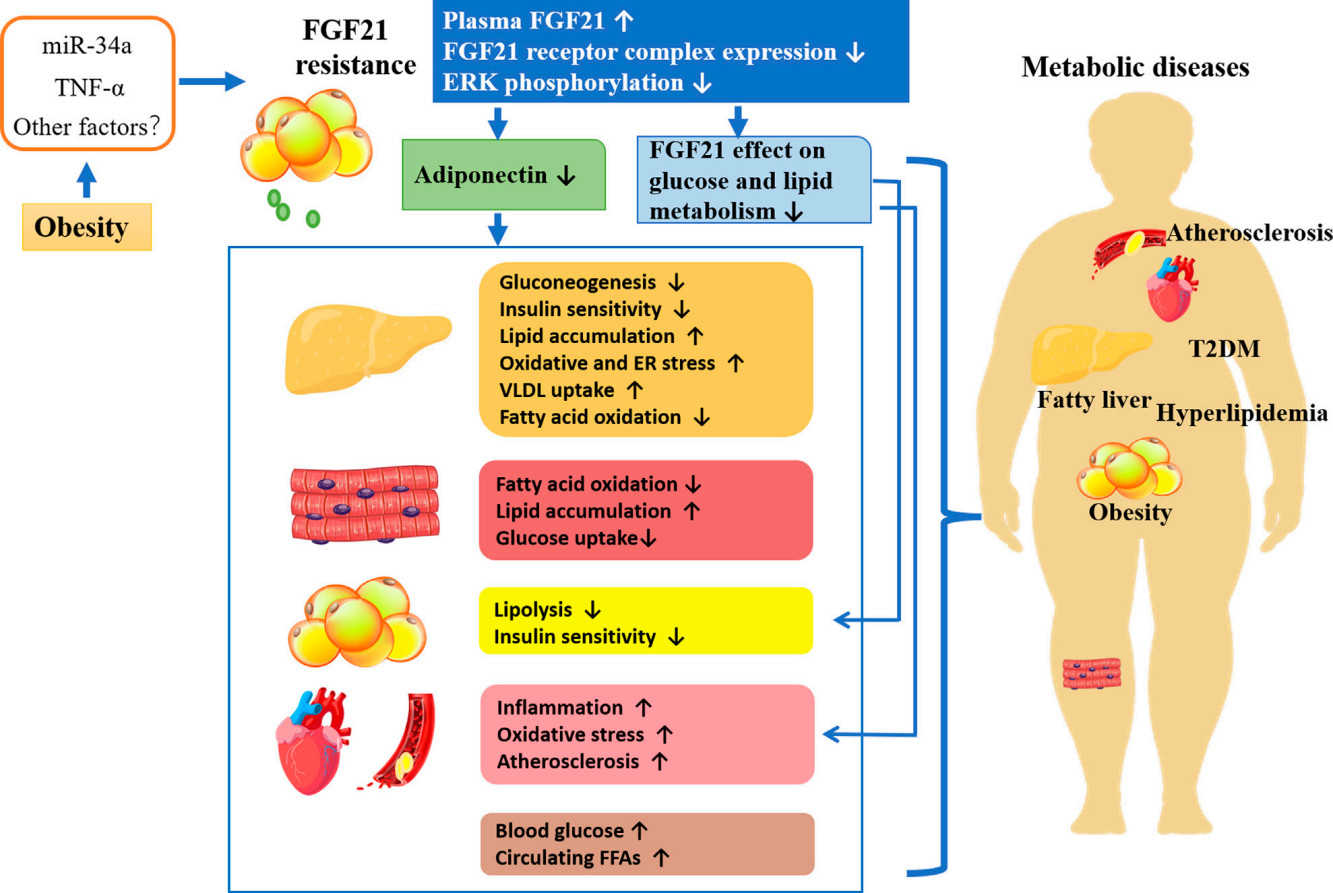
FGF21 resistance, the potential mechanism leading to metabolic diseases.

As mentioned previously, circulating FGF21 levels are elevated in obese animals and humans, suggesting the existence of FGF21 resistance. It is considered that FGF21 resistance may be the result of reduced expression of the FGF21 receptor complex. As shown in Figure 2, in obese patients, the secretion of proinflammatory factors and microRNAs or other factors induced by excess fat is increased. For example, TNF-alpha repressed KLB expression and impaired FGF21 action in adipocytes by activation of Jun N- terminal (JNK1) (Diaz-Delfin et al., 2012). Increased miR-34a in the adipose tissues of obese mice decreases KLB expression either directly by targeting the 3 ′-untranslated region of β-klotho or indirectly by inducing secretion of proinflammatory factors in adipose tissue (Fu et al., 2014; Pan et al., 2019). Obesity also led to a decrease in ERK1/2 phosphorylation (Fisher et al., 2010). Thus, FGF21 resistance leads to the impairment of FGF21 signaling cascade as well as reduced adiponectin secretion. Additionally, the ability of FGF21 acts directly on adipose tissue, and cardiovascular regulation of glucose and lipid metabolism is also impaired (Yang et al., 2019). Studies have shown that the FGF21-adiponectin axis plays an important role in systemic metabolism, and FGF21 acts indirectly on the liver and cardiovascular system by inducing adiponectin secretion (Hui et al., 2016). Overall, obesity-induced FGF21 resistance induces various metabolic diseases by impairing the direct effects of FGF21 and the indirect effects mediated by adiponectin or other factors leading to metabolic disorders, which might account for the explanation of the elevated FGF21 level in metabolic diseases.

However, the administration of exogenous FGF21 or its receptor agonist was approved to ultimately overcome FGF21 resistance and improve the effects in target organs related to metabolic homeostasis. Although the regulatory mechanism is not clear by far, pharmacological strategies, administration of exogenous FGF21 or its receptor agonist, keep developing, due to the restoration of FGF21 effect in target organs. During the last decades, the pleiotropic beneficial actions of FGF21 on metabolic disorders in animals promoted FGF21-based drugs for therapeutic purposes. However, native FGF21 has a brief circulatory half-life (0.5–2 h) due to proteolytic cleavage and rapid renal clearance. Thus, this has led to the development of FGF21 analogs and FGF21-receptor agonists. The update on recent pharmaceutical development of FGF21-based drugs for metabolic diseases, including their effects and current clinical trial status was summarized in Table 1.

**TABLE 1:** Pharmaceutical development of FGF21-based drugs for metabolic diseases.

Brand name	Company	Metabolic diseases		Study species	Beneficial effects	Clinical trial status
**FGF21 analogs**						
LY2405319	Eli Lilly	Obese; T2D		Human, rhesus, mice	Reduced plasma lipid, lipoproteins and fasting insulin level, increased HDL-c	Phase IIa
Pegbelfermin (BMS-986036)	Ambrx/Bristol-Myers Squibb	Obese; T2D; NASH		Human, mice	Increased HDL-C, adiponectin and whole-body insulin sensitivity, reduced LDL-C, fasting TG, fasting glucose, insulin levels and hepatic fat fraction	Phase IIb
Efruxifermin (AKR-001)	Akero	T2D; NASH		Human, mice	Increased HDL-C and adiponectin, decreased TG, improved glycemic control and markers of insulin sensitivity	Phase IIb
PF-05231023	Pfizer	Obese; T2D; hypertriglyceridemia		Human, cynomolgus, mice	Reduced LDL-C, fasting glucose, TG, glucose and insulin level, increased HDL-C, adiponectin and whole-body insulin sensitivity	—
BIO89-100	89 bio	NASH or phenotypic NASH		Human, cynomolgus	Reduced liver fat, ALT, PRO-C3, and MRI-PDFF, increased adiponectin	Phase IIa
B1344	Tasly Biopharma	NAFLD; NASH		Cynomolgus, mice	Reduced body weight, liver fat, ALT, AST, TG, fasting glucose, IL1β, MCP1, TNFα, CD68, F4/80, HbA1c, VLDL, increased adiponectin, improved plasma lipid profile	Phase I
**FGF21-receptor agonists**						
C3201–HSA	Amgen Inc.	Obese		Cynomolgus	Reduced body weight, fasting plasma insulin levels and TC	—
MimAb1; 39F7mAb	Amgen Inc.	Obese		Cynomolgus, mice	Reduced body weight, BMI, plasma glucose and insulin level, TC, TG	—
MK-3655 (NGM313)	Merck Sharp & Dohme	NAFLD		Human	Reduced liver fat, HbA1c, insulin resistance and transaminase, improved dyslipidemia	Phase IIb
BFKB8488A	Genentech	NAFLD		Human, cynomolgus	Reduced body weight, food intake, LDL-C, TG, increased HDL-C, adiponectin	Phase IIb

### 3.1 FGF21 analogs

#### 3.1.1 LY2405319

LY2405319, an FGF21 analog developed by Lilly Research Laboratories in 2013, has shown the same efficacy and biological activity as native human FGF21 (Adams et al., 2013; Gaich et al., 2013). It has an additional engineered disulfide bond at Leu118Cys-Ala134Cys stabilized a loop at the C-terminal domain of FGF21 introduced *via* point-specific mutagenesis (Kharitonenkov et al., 2013).

In a 28-day trial, the safety and tolerability of LY2405319 were shown in obese subjects with T2DM. The four Lipid parameters (total cholesterol, LDL, HDL, and TGs) and body weight showed statistically different compared to baseline, which was consistent with the results of FGF21 or LY2405319 administration to obese rhesus monkeys with dyslipidemia (Kharitonenkov et al., 2007; Adams et al., 2013; Gaich et al., 2013). The impact of LY2405319 treatment on fasting TGs was rapid, accompanied by reduced plasma ApoCIII and ApoB (Gaich et al., 2013). Body weight was also reduced by LY2405319 over the 28-day treatment, which coincided with the increase in plasma β-hydroxybutyrate. This suggests fatty acid oxidation is enhanced by the analog similar to the findings in rodents (Coskun et al., 2008; Fisher et al., 2011; Gaich et al., 2013). At the same time, the mean fasting insulin level decreased and the plasma adiponectin level increased substantially in a dose-dependent manner (Gaich et al., 2013).

LY2405319 has also been proven effective in suppressing liver inflammation and fibrosis in preclinical experiments. A study showed that FGF21 reduced α-SMA production by inhibiting succinate -GPR91 signaling in HSCs and improved hepatic steatosis and fibrosis in an MCD diet-induced mouse model (Le CT et al., 2018). Another study found that LY2405319 improved metabolic parameters and symptoms of steatohepatitis by increasing oxygen consumption rate and fatty acid oxidation in muscle mitochondria (Le CT et al., 2018). In addition, LY2405319 significantly reduced the serum AST and ALT levels and decreased the expression of pro-fibrosis markers TGF-β1 and collagen I, suggesting that liver injury was alleviated (Lee et al., 2016).

#### 3.1.2 Pegbelfermin (BMS-986036)

Pegbelfermin (PGBF), a polyethylene glycol (PEG)-conjugated recombinant analog of human FGF21, has a prolonged half-life that supports up to weekly dosing.

In phase 2 clinical study, PGBF was proved safe and well tolerated in patients with obesity, T2DM treated daily or weekly for 12 weeks. The treatment significantly improved the serum HDL, TGs, adiponectin, and Pro-C3 of the participants (Charles et al., 2019).

In another phase IIa trial, patients with stage 1–3 of NASH were treated with PGBF at a dose of 10 mg daily or 20 mg weekly subcutaneous injection for 16 weeks. The results showed beneficial effects of PGBF on NASH, including decreased liver fat fraction and improved metabolic parameters (blood adiponectin and lipid concentrations), markers of liver injury (ALT and AST), and fibrosis biomarkers (liver stiffness and Pro-C3) (Sanyal et al., 2019). Further studies showed that PGBF selectively downregulated serum levels of deoxycholic acid (DCA) and conjugates in NASH patients and suggest PGBF can modulate secondary BA synthesis, which may contribute to the role of PGBF against NASH (Luo et al., 2022). Furthermore, the FALCON phase IIb studies currently in progress are to investigate the efficacy and safety of PGBF specifically over a longer period (48 weeks) in patients with NASH and advanced fibrosis with stage 3 fibrosis (FALCON1) or compensated cirrhosis (FALCON2), who are at the highest risk of decompensated liver disease and HCC (Abdelmalek et al., 2021).

#### 3.1.3 Efruxifermin (AKR-001)

Efruxifermin is a fusion protein of the human IgG1 Fc domain linked to a modified human FGF21 (Fc-FGF21) with a 3–3.5-day half-life. It has equal *in vitro* agonist potency for FGFR1c, FGFR2c, and FGFR3c (Stanislaus et al., 2017). The results from a phase I studies in individuals with T2D showed improvements in glycemic control and lipoprotein profiles, including triglycerides, cholesterol, and apolipoproteins B and C3 (Kaufman et al., 2020).

In addition, a randomized, placebo-controlled phase IIa trial (BALANCED) study in patients with NASH *via* weekly subcutaneous administration of Efruxifermin for 16 weeks demonstrated that Efruxifermin significantly reduced hepatic fat fraction (HFF) in patients with F1-F3 NASH (Harrison et al., 2021).

#### 3.1.4 PF-05231023

PF-05231023 is a long-acting FGF21 analog. It has been shown in rodents to reduce body weight and improve glucose tolerance (Weng et al., 2015). Another study in obese non-human primates showed that PF-05231023 reduced body weight only by lowering food intake and had no direct effect on energy expenditure (Thompson et al., 2016). In a placebo-controlled study of overweight/obese patients with T2D, PF-05231023 reduced body weight and improved plasma lipoprotein profiles and adiponectin levels significantly, with no obvious effect on glycemic control (Talukdar et al., 2016b). These studies support this molecule for the treatment of obesity.

However, in obese patients with high triglycerides with and without T2DM, PF-05231023 administration once a week significantly reduced serum TG level without weight loss. However, adverse changes that systolic blood pressure, diastolic blood pressure, and pulse rate were found increased by PF-05231023 in a dose- and time-dependent manner (Kim et al., 2017).

#### 3.1.5 Pegozafermin (BIO89-100)

Pegozafermin is another long-acting glycol PEGylated FGF21 analog, which is currently the only one with the potential of use once every 2 weeks dosing. Recently, a phase Ib/IIa trial enrolled and randomized NASH subjects with liver fat ≥10% to 12 weeks of treatment with BIO89-100 or placebo. The results showed decreased liver fat in all BIO89-100 groups, accompanied by significant decreases in ALT, and Pro-C3, along with increases in adiponectin levels observed (89bio, 2022a). There were 63% of patients achieved NAS score improvement by more than two points without deteriorated fibrosis, and 74% of patients achieved NAS score improvement. Additionally, no adverse effects associated with deaths, blood pressure, or heart rate were reported (89bio, 2022b).

#### 3.1.6 B1344

B1344 is a recombinant PEGylatedhuman FGF21 protein. The safety and efficacy of B1344 in the treatment of non-human primate or rodent NAFLD models have been investigated. B1344 subcutaneous injection for 11 weeks remarkably reduced body weight and improved the degree of steatosis, inflammation, and fibrosis of liver tissues, lipid profiles, and glycemic control of cynomolgus monkeys with NAFLD (Cui et al., 2020). Consistently, improvement of lipotoxic injury by B1344 was also observed in the MCD diet-induced NASH model of mice (Cui et al., 2020). Moreover, in our recent study, B1344 administration to NASH model of mice fed an HFD-high fructose diet twice per week for 8 weeks obviously ameliorated the liver fibrosis degree in addition to the reduction of other metabolism-related parameters. Recently, clinical trials of B1344 were approved for the treatment of NASH by FDA and for the treatment of T2D by China State Drug Administration.

### 3.2 FGF21-receptor agonists (FGFRAs)

#### 3.2.1 C3201-HSA

The first FGF21receptor agonist is C3201, an 18 kDa bispecific avimer peptide with high affinity and specificity for the FGFR1-KLB receptor complex. Fusion with human serum albumin forms C3201-HAS, which has a longer half-life of about 50 h. It has been found that C3201-HAS could trigger the effects of FGF21 in obese monkeys, reducing body weight, fasting insulin concentrations, and plasma TG levels (Smith et al., 2013).

#### 3.2.2 MimAb1 and 39F7mAb

Two fully humanized FGF21-mimetic monoclonal agonist antibodies (mAbs) (mimAb1 and 39F7 mAb) for FGF21 receptor were developed by Amgen Inc. They can bind to distinct conformational epitopes of KLB with high affinity and specificity, activate the receptor complex, and drive downstream signaling. Administraion of mimab1 in obese monkeys led to FGF21-like metabolic regulatory effects, including body weight loss and improvement in plasma insulin, lipid, and glucose levels (Foltz et al., 2012).

#### 3.2.3 MK-3665 (NGM313)

Recently, Merck Sharp & Dohme have developed a monthly used antibody MK-3655 (previous name NGM313) that can activate the FGF21 receptor complex. Clinical trials have proved that treatment of MK-3665 once every 36 days reduced liver steatosis degree, improve hyperlipidemia, and decrease HbA1c level and transaminases in patients with obesity, IR, and NAFLD (Depaoli et al., 2019).

#### 3.2.4 BFKB8488A

BFKB8488A is a full-length, humanized bispecific antiFGFR1c/KLB agonist antibody. It can selectively activate FGFR1 in a KLB-dependent manner and mimics the FGF21function. It has been demonstrated in a randomized study, improvement in body weight, cardiometabolic parameters, and reduced preference for carbohydrate intake and sweet taste in overweight/obese human with BFKB8488A administration. (Baruch et al., 2020). In a Phase Ib clinical trial, the efficacy was also demonstrated with improved parameters observed in patients with both T2D and NAFLD, especially with a dose- and time-dependent reduction of hepatosteatosis in patients with NAFLD (Wong et al., 2022). Therefore, Genentech recently initiated a Phase 2b (BANFF) clinical trial to evaluate the efficacy, safety, and pharmacokinetics of the drug in NASH patients with F2-3 fibrosis score.

## 4 Conclusion

The rapid increasing prevalence of metabolic syndrome, T2DM, and NASH constitutes great burden on global public health, which need effective intervention to prevent them from developing into serious diseases like cardiovascular disease, stroke, and liver cirrhosis, *etc.* Emerging evidence demonstrates that circulating FGF21 can be used as a predictor or biomarker of some metabolic diseases such as diabetes, and CAD, because of its aberrantly increasing level. More importantly, FGF21-based drugs are explored to ameliorate metabolic diseases because of their crucial actions in regulating systemic glucose and lipid metabolism. And during past decades, the beneficial effect of FGF21 analogs and FGF21-receptor agonists confirmed by preclinical and clinical experiments has indicated that FGF21 is an attractive target for the treatment of metabolic diseases, particularly for obesity, T2DM, and NASH.

However, the safety associated with FGF21-based drugs still warrants further research. The adverse effect of FGF21 treatment such as reduced fertility in female mice (Owen et al., 2013), increased plasma corticosterone (Bookout et al., 2013), and impaired bone mineral density has been observed (Wei et al., 2012). But there are also inconsistent results. In clinical trials, obese subjects receiving PF05231023 treatment showed changes in bone markers (Talukdar et al., 2016a), whereas no changes in bone density were observed in patients with Pegbelfermin treatment (Sanyal et al., 2019). The effect of FGF21 on female fertility may be related to relatively substantial weight loss (Coskun et al., 2008), which may be overcome by intaking an HFD to increase the kisspeptin expression (Singhal et al., 2016a). Previous studies considered FGF21 had less risk of cancer induction since it is the only family member without mitogenic action. However, aberrant expression of FGF21 has been found related to cancer development, and it is suggested as a promising cancer biomarker (Sui and Chen, 2022).

Therefore, further understanding of the mechanisms involved in the metabolic regulation attributed to targeting FGF21 is necessary, which will facilitate the development of more effective and safer drugs for the treatment of metabolic diseases.

## References

[B1] 89bio (2022b). Pegozafermin (BIO89-100) phase 1b/2a NASH histology cohort topline results. Available at: https://ir.89bio.com/static-files/4046a1f3-c63d-40aa-ba18-af4f36711a9e.

[B2] 89bio (2022a). Reports positive topline results from an expansion cohort of the phase 1b/2a trial of pegozafermin (BIO89-100) for the treatment of NASH. Available: https://ir.89bio.com/news-releases/news-release-details/89bio-reports-positive-topline-results-expansion-cohort-phase 2022 .

[B3] Abdelmalek M. F. Charles E. D. Sanyal A. J. Harrison S. A. Neuschwander-Tetri B. A. Goodman Z. (2021). The FALCON program: Two phase 2b randomized, double-blind, placebo-controlled studies to assess the efficacy and safety of pegbelfermin in the treatment of patients with nonalcoholic steatohepatitis and bridging fibrosis or compensated cirrhosis. Contemp. Clin. Trials. 104, 106335. 10.1016/j.cct.2021.106335 33657443

[B4] Adams A. C. Halstead C. A. Hansen B. C. Irizarry A. R. Martin J. A. Myers S. R. (2013). LY2405319, an engineered FGF21 variant, improves the metabolic status of diabetic monkeys. PLoS One 8 (6), e65763. 10.1371/journal.pone.0065763 23823755 PMC3688819

[B5] Attili A. F. Angelico M. Cantafora A. Alvaro D. Capocaccia L. (1986). Bile acid-induced liver toxicity: Relation to the hydrophobic-hydrophilic balance of bile acids. Med. Hypotheses. 19 (1), 57–69. 10.1016/0306-9877(86)90137-4 2871479

[B6] Badman M. K. Koester A. Flier J. S. Kharitonenkov A. Maratos-Flier E. (2009). Fibroblast growth factor 21-deficient mice demonstrate impaired adaptation to ketosis. Endocrinology 150 (11), 4931–4940. 10.1210/en.2009-0532 19819944 PMC2775979

[B7] Badman M. K. Pissios P. Kennedy A. R. Koukos G. Flier J. S. Maratos-Flier E. (2007). Hepatic fibroblast growth factor 21 is regulated by PPARalpha and is a key mediator of hepatic lipid metabolism in ketotic states. Cell Metab. 5 (6), 426–437. 10.1016/j.cmet.2007.05.002 17550778

[B8] Bao L. Yin J. Gao W. Wang Q. Yao W. Gao X. (2018). A long-acting FGF21 alleviates hepatic steatosis and inflammation in a mouse model of non-alcoholic steatohepatitis partly through an FGF21-adiponectin-IL17A pathway. Br. J. Pharmacol. 175 (16), 3379–3393. 10.1111/bph.14383 29859019 PMC6057909

[B9] Barbera M. J. Schluter A. Pedraza N. Iglesias R. Villarroya F. Giralt M. (2001). Peroxisome proliferator-activated receptor alpha activates transcription of the Brown fat uncoupling protein-1 gene. A link between regulation of the thermogenic and lipid oxidation pathways in the Brown fat cell. J. Biol. Chem. 276 (2), 1486–1493. 10.1074/jbc.M006246200 11050084

[B10] Bartelt A. Bruns O. T. Reimer R. Hohenberg H. Ittrich H. Peldschus K. (2011). Brown adipose tissue activity controls triglyceride clearance. Nat. Med. 17 (2), 200–205. 10.1038/nm.2297 21258337

[B11] Baruch A. Wong C. Chinn L. W. Vaze A. Sonoda J. Gelzleichter T. (2020). Antibody-mediated activation of the FGFR1/Klothoβ complex corrects metabolic dysfunction and alters food preference in obese humans. Proc. Natl. Acad. Sci. U. S. A. 117 (46), 28992–29000. 10.1073/pnas.2012073117 33139537 PMC7682391

[B12] Beenken A. Mohammadi M. (2009). The FGF family: Biology, pathophysiology and therapy. Nat. Rev. Drug Discov. 8 (3), 235–253. 10.1038/nrd2792 19247306 PMC3684054

[B13] Bondurant L. D. Ameka M. Naber M. C. Markan K. R. Idiga S. O. Acevedo M. R. (2017). FGF21 regulates metabolism through adipose-dependent and -independent mechanisms. Cell Metab. 25 (4), 935–944. e4. 10.1016/j.cmet.2017.03.005 28380381 PMC5494834

[B14] Bookout A. L. de Groot M. H. Owen B. M. Lee S. Gautron L. Lawrence H. L. (2013). FGF21 regulates metabolism and circadian behavior by acting on the nervous system. Nat. Med. 19 (9), 1147–1152. 10.1038/nm.3249 23933984 PMC3769420

[B15] Cancilla B. Davies A. Cauchi J. A. Risbridger G. P. Bertram J. F. (2001). Fibroblast growth factor receptors and their ligands in the adult rat kidney. Kidney Int. 60 (1), 147–155. 10.1046/j.1523-1755.2001.00781.x 11422746

[B16] Cancilla B. Ford-Perriss M. D. Bertram J. F. (1999). Expression and localization of fibroblast growth factors and fibroblast growth factor receptors in the developing rat kidney. Kidney Int. 56 (6), 2025–2039. 10.1046/j.1523-1755.1999.00781.x 10594778

[B17] Cannon B. Nedergaard J. (2004). Brown adipose tissue: Function and physiological significance. Physiol. Rev. 84 (1), 277–359. 10.1152/physrev.00015.2003 14715917

[B18] Charles E. D. Neuschwander Tetri B. A. Pablo Frias J. Kundu S. Luo Y. Tirucherai G. S. (2019). Pegbelfermin (BMS‐986036), PEGylated FGF21, in patients with obesity and type 2 diabetes: Results from a randomized phase 2 study. Obes. (Silver Spring, Md 27 (1), 41–49. 10.1002/oby.22344 PMC6587787 30520566

[B19] Chartoumpekis D. V. Habeos I. G. Ziros P. G. Psyrogiannis A. I. Kyriazopoulou V. E. Papavassiliou A. G. (2011). Brown adipose tissue responds to cold and adrenergic stimulation by induction of FGF21. Mol. Med. 17 (7-8), 736–740. 10.2119/molmed.2011.00075 21373720 PMC3146611

[B20] Chau M. D. Gao J. Yang Q. Wu Z. Gromada J. (2010). Fibroblast growth factor 21 regulates energy metabolism by activating the AMPK-SIRT1-PGC-1alpha pathway. Proc. Natl. Acad. Sci. U. S. A. 107 (28), 12553–12558. 10.1073/pnas.1006962107 20616029 PMC2906565

[B21] Chen K. Rao Z. Dong S. Chen Y. Wang X. Luo Y. (2022). Roles of the fibroblast growth factor signal transduction system in tissue injury repair. Burns Trauma 10, tkac005. 10.1093/burnst/tkac005 35350443 PMC8946634

[B22] Chen M. M. Hale C. Stanislaus S. Xu J. Véniant M. M. (2018). FGF21 acts as a negative regulator of bile acid synthesis. J. Endocrinol. 237 (2), 139–152. 10.1530/JOE-17-0727 29615519

[B23] Coskun T. Bina H. A. Schneider M. A. Dunbar J. D. Hu C. C. Chen Y. (2008). Fibroblast growth factor 21 corrects obesity in mice. Endocrinology 149 (12), 6018–6027. 10.1210/en.2008-0816 18687777

[B24] Cui A. Li J. Ji S. Ma F. Wang G. Xue Y. (2020). The effects of B1344, a novel fibroblast growth factor 21 analog, on nonalcoholic steatohepatitis in nonhuman primates. Diabetes 69 (8), 1611–1623. 10.2337/db20-0209 32354858

[B25] Cummings N. E. Williams E. M. Kasza I. Konon E. N. Schaid M. D. Schmidt B. A. (2018). Restoration of metabolic health by decreased consumption of branched-chain amino acids. J. Physiol. 596 (4), 623–645. 10.1113/JP275075 29266268 PMC5813603

[B26] Cyphert H. A. Alonge K. M. Ippagunta S. M. Hillgartner F. B. (2014). Glucagon stimulates hepatic FGF21 secretion through a PKA- and EPAC-dependent posttranscriptional mechanism. PLoS One 9 (4), e94996. 10.1371/journal.pone.0094996 24733293 PMC3986400

[B27] Cyphert H. A. Ge X. Kohan A. B. Salati L. M. Zhang Y. Hillgartner F. B. (2012). Activation of the farnesoid X receptor induces hepatic expression and secretion of fibroblast growth factor 21. J. Biol. Chem. 287 (30), 25123–25138. 10.1074/jbc.M112.375907 22661717 PMC3408207

[B28] Depaoli A. Phung V. Bashir M. R. Morrow L. Beysen C. Yan A. (2019). 140-LB: NGM313, a novel activator of b-klotho/FGFR1c, improves insulin resistance and reduces hepatic fat in obese, nondiabetic subjects. Diabetes 68 (1), 140. 10.2337/db19-140-lb

[B29] Diaz-Delfin J. Hondares E. Iglesias R. Giralt M. Caelles C. Villarroya F. (2012). TNF-Alpha represses beta-klotho expression and impairs FGF21 action in adipose cells: Involvement of JNK1 in the FGF21 pathway. Endocrinology 153 (9), 4238–4245. 10.1210/en.2012-1193 22778214

[B30] Dutchak P. A. Katafuchi T. Bookout A. L. Choi J. H. Yu R. T. Mangelsdorf D. J. (2012). Fibroblast growth factor-21 regulates PPARγ activity and the antidiabetic actions of thiazolidinediones. Cell 148 (3), 556–567. 10.1016/j.cell.2011.11.062 22304921 PMC3273727

[B31] Fazeli P. K. Lun M. Kim S. M. Bredella M. A. Wright S. Zhang Y. (2015). FGF21 and the late adaptive response to starvation in humans. J. Clin. Invest. 125 (12), 4601–4611. 10.1172/JCI83349 26529252 PMC4665770

[B32] Fisher F. M. Chui P. C. Antonellis P. J. Bina H. A. Kharitonenkov A. Flier J. S. (2010). Obesity is a fibroblast growth factor 21 (FGF21)-resistant state. Diabetes 59 (11), 2781–2789. 10.2337/db10-0193 20682689 PMC2963536

[B33] Fisher F. M. Chui P. C. Nasser I. A. Popov Y. Cunniff J. C. Lundasen T. (2014). Fibroblast growth factor 21 limits lipotoxicity by promoting hepatic fatty acid activation in mice on methionine and choline-deficient diets. Gastroenterology 147 (5), 1073–1083. e6. 10.1053/j.gastro.2014.07.044 25083607 PMC4570569

[B34] Fisher F. M. Estall J. L. Adams A. C. Antonellis P. J. Bina H. A. Flier J. S. (2011). Integrated regulation of hepatic metabolism by fibroblast growth factor 21 (FGF21) *in vivo* . Endocrinology 152 (8), 2996–3004. 10.1210/en.2011-0281 21712364 PMC3138239

[B35] Fisher F. M. Kim M. Doridot L. Cunniff J. C. Parker T. S. Levine D. M. (2017). A critical role for ChREBP-mediated FGF21 secretion in hepatic fructose metabolism. Mol. Metab. 6 (1), 14–21. 10.1016/j.molmet.2016.11.008 28123933 PMC5220398

[B36] Fisher F. M. Kleiner S. Douris N. Fox E. C. Mepani R. J. Verdeguer F. (2012). FGF21 regulates PGC-1α and browning of white adipose tissues in adaptive thermogenesis. Gene. Dev. 26 (3), 271–281. 10.1101/gad.177857.111 22302939 PMC3278894

[B37] Flippo K. H. Potthoff M. J. (2021). Metabolic messengers: FGF21. Nat. Metab. 3 (3), 309–317. 10.1038/s42255-021-00354-2 33758421 PMC8620721

[B38] Foltz I. N. Hu S. King C. Wu X. Yang C. Wang W. (2012). Treating diabetes and obesity with an FGF21-mimetic antibody activating the βKlotho/FGFR1c receptor complex. Sci. Transl. Med. 4 (162), 162ra153. 10.1126/scitranslmed.3004690 23197570

[B39] Fon Tacer K. Bookout A. L. Ding X. Kurosu H. John G. B. Wang L. (2010). Research resource: Comprehensive expression atlas of the fibroblast growth factor system in adult mouse. Mol. Endocrinol. Baltim. Md 24 (10), 2050–2064. 10.1210/me.2010-0142 PMC2954642 20667984

[B40] Fu T. Seok S. Choi S. Huang Z. Suino-Powell K. Xu H. E. (2014). MicroRNA 34a inhibits beige and Brown fat formation in obesity in part by suppressing adipocyte fibroblast growth factor 21 signaling and SIRT1 function. Mol. Cell. Biol. 34 (22), 4130–4142. 10.1128/MCB.00596-14 25182532 PMC4248715

[B41] Gaich G. Chien J. Y. Fu H. Glass L. C. Deeg M. A. Holland W. L. (2013). The effects of LY2405319, an FGF21 analog, in obese human subjects with type 2 diabetes. Cell Metab. 18 (3), 333–340. 10.1016/j.cmet.2013.08.005 24011069

[B42] Gavalda-Navarro A. Hondares E. Giralt M. Mampel T. Iglesias R. Villarroya F. (2015). Fibroblast growth factor 21 in breast milk controls neonatal intestine function. Sci. Rep. 5, 13717. 10.1038/srep13717 26329882 PMC4557064

[B43] Geidl-Flueck B. Hochuli M. Spinas G. A. Gerber P. A. (2022). Do sugar-sweetened beverages increase fasting FGF21 irrespective of the type of added sugar? A secondary exploratory analysis of a randomized controlled trial. Nutrients 14 (19), 4169. 10.3390/nu14194169 36235821 PMC9572320

[B44] Geng L. Liao B. Jin L. Huang Z. Triggle C. R. Ding H. (2019). Exercise alleviates obesity-induced metabolic dysfunction via enhancing FGF21 sensitivity in adipose tissues. Cell Rep. 26 (10), 2738–2752. e4. 10.1016/j.celrep.2019.02.014 30840894

[B45] Goetz R. Beenken A. Ibrahimi O. A. Kalinina J. Olsen S. K. Eliseenkova A. V. (2007). Molecular insights into the klotho-dependent, endocrine mode of action of fibroblast growth factor 19 subfamily members. Mol. Cell. Biol. 27 (9), 3417–3428. 10.1128/MCB.02249-06 17339340 PMC1899957

[B46] Hansen J. S. Clemmesen J. O. Secher N. H. Hoene M. Drescher A. Weigert C. (2015). Glucagon-to-insulin ratio is pivotal for splanchnic regulation of FGF-21 in humans. Mol. Metab. 4 (8), 551–560. 10.1016/j.molmet.2015.06.001 26266087 PMC4529499

[B47] Harrison S. A. Ruane P. J. Freilich B. L. Neff G. Patil R. Behling C. A. (2021). Efruxifermin in non-alcoholic steatohepatitis: A randomized, double-blind, placebo-controlled, phase 2a trial. Nat. Med. 27 (7), 1262–1271. 10.1038/s41591-021-01425-3 34239138

[B48] Hayden M. S. West A. P. Ghosh S. (2006). NF-kappaB and the immune response. Oncogene 25 (51), 6758–6780. 10.1038/sj.onc.1209943 17072327

[B49] Hill C. M. Laeger T. Dehner M. Albarado D. C. Clarke B. Wanders D. (2019). FGF21 signals protein status to the brain and adaptively regulates food choice and metabolism. Cell Rep. 27 (10), 2934–2947. e3. 10.1016/j.celrep.2019.05.022 31167139 PMC6579533

[B50] Hondares E. Iglesias R. Giralt A. Gonzalez F. J. Giralt M. Mampel T. (2011). Thermogenic activation induces FGF21 expression and release in Brown adipose tissue. J. Biol. Chem. 286 (15), 12983–12990. 10.1074/jbc.M110.215889 21317437 PMC3075644

[B51] Hsuchou H. Pan W. Kastin A. J. (2007). The fasting polypeptide FGF21 can enter brain from blood. Peptides 28 (12), 2382–2386. 10.1016/j.peptides.2007.10.007 17996984 PMC2151924

[B52] Hui X. Feng T. Liu Q. Gao Y. Xu A. (2016). The FGF21-adiponectin axis in controlling energy and vascular homeostasis. J. Mol. Cell Biol. 8 (2), 110–119. 10.1093/jmcb/mjw013 26993043

[B53] Inagaki T. Dutchak P. Zhao G. Ding X. Gautron L. Parameswara V. (2007). Endocrine regulation of the fasting response by PPARalpha-mediated induction of fibroblast growth factor 21. Cell Metab. 5 (6), 415–425. 10.1016/j.cmet.2007.05.003 17550777

[B54] Ito S. Kinoshita S. Shiraishi N. Nakagawa S. Sekine S. Fujimori T. (2000). Molecular cloning and expression analyses of mouse betaklotho, which encodes a novel Klotho family protein. Mech. Dev. 98 (1-2), 115–119. 10.1016/s0925-4773(00)00439-1 11044614

[B55] Itoh N. Ornitz D. M. (2011). Fibroblast growth factors: From molecular evolution to roles in development, metabolism and disease. J. Biochem. 149 (2), 121–130. 10.1093/jb/mvq121 20940169 PMC3106964

[B56] Jensen-Cody S. O. Flippo K. H. Claflin K. E. Yavuz Y. Sapouckey S. A. Walters G. C. (2020). FGF21 signals to glutamatergic neurons in the ventromedial hypothalamus to suppress carbohydrate intake. Cell Metab. 32 (2), 273–286. e6. 10.1016/j.cmet.2020.06.008 32640184 PMC7734879

[B57] Jiang S. Yan C. Fang Q. C. Shao M. L. Zhang Y. L. Liu Y. (2014). Fibroblast growth factor 21 is regulated by the IRE1α-XBP1 branch of the unfolded protein response and counteracts endoplasmic reticulum stress-induced hepatic steatosis. J. Biol. Chem. 289 (43), 29751–29765. 10.1074/jbc.M114.565960 25170079 PMC4207989

[B58] Jimenez V. Jambrina C. Casana E. Sacristan V. Munoz S. Darriba S. (2018). FGF21 gene therapy as treatment for obesity and insulin resistance. EMBO Mol. Med. 10 (8), e8791. 10.15252/emmm.201708791 29987000 PMC6079533

[B59] Johnson C. L. Mehmood R. Laing S. W. Stepniak C. V. Kharitonenkov A. Pin C. L. (2014). Silencing of the Fibroblast growth factor 21 gene is an underlying cause of acinar cell injury in mice lacking MIST1. Am. J. Physiol. Endocrinol. Metab. 306 (8), E916–E928. 10.1152/ajpendo.00559.2013 24549397

[B60] Johnson C. L. Weston J. Y. Chadi S. A. Fazio E. N. Huff M. W. Kharitonenkov A. (2009). Fibroblast growth factor 21 reduces the severity of cerulein-induced pancreatitis in mice. Gastroenterology 137 (5), 1795–1804. 10.1053/j.gastro.2009.07.064 19664632

[B61] Joki Y. Ohashi K. Yuasa D. Shibata R. Ito M. Matsuo K. (2015). FGF21 attenuates pathological myocardial remodeling following myocardial infarction through the adiponectin-dependent mechanism. Biochem. Biophys. Res. Commun. 459 (1), 124–130. 10.1016/j.bbrc.2015.02.081 25712519

[B62] Kaufman A. Abuqayyas L. Denney W. S. Tillman E. J. Rolph T. (2020). AKR-001, an fc-FGF21 analog, showed sustained pharmacodynamic effects on insulin sensitivity and lipid metabolism in type 2 diabetes patients. Cell Rep. Med. 1 (4), 100057. 10.1016/j.xcrm.2020.100057 33205064 PMC7659583

[B63] Keipert S. Kutschke M. Lamp D. Brachthauser L. Neff F. Meyer C. W. (2015). Genetic disruption of uncoupling protein 1 in mice renders Brown adipose tissue a significant source of FGF21 secretion. Mol. Metab. 4 (7), 537–542. 10.1016/j.molmet.2015.04.006 26137441 PMC4481421

[B64] Keipert S. Lutter D. Schroeder B. O. Brandt D. Stahlman M. Schwarzmayr T. (2020). Endogenous FGF21-signaling controls paradoxical obesity resistance of UCP1-deficient mice. Nat. Commun. 11 (1), 624. 10.1038/s41467-019-14069-2 32005798 PMC6994690

[B65] Kharitonenkov A. Adams A. C. (2014). Inventing new medicines: The FGF21 story. Mol. Metab. 3 (3), 221–229. 10.1016/j.molmet.2013.12.003 24749049 PMC3986619

[B66] Kharitonenkov A. Beals J. M. Micanovic R. Strifler B. A. Rathnachalam R. Wroblewski V. J. (2013). Rational design of a fibroblast growth factor 21-based clinical candidate, LY2405319. PLoS One 8 (3), e58575. 10.1371/journal.pone.0058575 23536797 PMC3594191

[B67] Kharitonenkov A. Shiyanova T. L. Koester A. Ford A. M. Micanovic R. Galbreath E. J. (2005). FGF-21 as a novel metabolic regulator. J. Clin. Invest. 115 (6), 1627–1635. 10.1172/JCI23606 15902306 PMC1088017

[B68] Kharitonenkov A. Wroblewski V. J. Koester A. Chen Y. F. Clutinger C. K. Tigno X. T. (2007). The metabolic state of diabetic monkeys is regulated by fibroblast growth factor-21. Endocrinology 148 (2), 774–781. 10.1210/en.2006-1168 17068132

[B69] Kim A. M. Somayaji V. R. Dong J. Q. Rolph T. P. Weng Y. Chabot J. R. (2017). Once-weekly administration of a long-acting fibroblast growth factor 21 analogue modulates lipids, bone turnover markers, blood pressure and body weight differently in obese people with hypertriglyceridaemia and in non-human primates. Diabetes Obes. Metab. 19 (12), 1762–1772. 10.1111/dom.13023 28573777

[B70] Kim H. W. Lee J. E. Cha J. J. Hyun Y. Y. Kim J. E. Lee M. H. (2013). Fibroblast growth factor 21 improves insulin resistance and ameliorates renal injury in db/db mice. Endocrinology 154 (9), 3366–3376. 10.1210/en.2012-2276 23825123

[B71] Kim S. H. Kim K. H. Kim H. Kim M. Back S. H. Konishi M. (2014). Fibroblast growth factor 21 participates in adaptation to endoplasmic reticulum stress and attenuates obesity-induced hepatic metabolic stress. Diabetologia 58 (4), 809–818. 10.1007/s00125-014-3475-6 25537833

[B72] Kurosu H. Choi M. Ogawa Y. Dickson A. S. Goetz R. Eliseenkova A. V. (2007). Tissue-specific expression of betaKlotho and fibroblast growth factor (FGF) receptor isoforms determines metabolic activity of FGF19 and FGF21. J. Biol. Chem. 282 (37), 26687–26695. 10.1074/jbc.M704165200 17623664 PMC2496965

[B73] Kwon M. M. O'Dwyer S. M. Baker R. K. Covey S. D. Kieffer T. J. (2015). FGF21-Mediated improvements in glucose clearance require uncoupling protein 1. Cell Rep. 13 (8), 1521–1527. 10.1016/j.celrep.2015.10.021 26586424

[B74] Larsson S. C. Michaëlsson K. Mola-Caminal M. Höijer J. Mantzoros C. S. (2022). Genome-wide association and Mendelian randomization study of fibroblast growth factor 21 reveals causal associations with hyperlipidemia and possibly NASH. Metabolism 137, 155329. 10.1016/j.metabol.2022.155329 36208799

[B75] Le C. T. Nguyen G. Park S. Y. Choi D. H. Cho E. H. (2018). LY2405319, an analog of fibroblast growth factor 21 ameliorates alpha-smooth muscle actin production through inhibition of the succinate-G-protein couple receptor 91 (GPR91) pathway in mice. PLoS One 13 (2), e0192146. 10.1371/journal.pone.0192146 29444136 PMC5812602

[B76] Lee J. H. Kang Y. E. Chang J. Y. Park K. C. Kim H. W. Kim J. T. (2016). An engineered FGF21 variant, LY2405319, can prevent non-alcoholic steatohepatitis by enhancing hepatic mitochondrial function. Am. J. Transl. Res. 8 (11), 4750–4763. 27904677 PMC5126319

[B77] Lee M. S. Choi S. E. Ha E. S. An S. Y. Kim T. H. Han S. J. (2012). Fibroblast growth factor-21 protects human skeletal muscle myotubes from palmitate-induced insulin resistance by inhibiting stress kinase and NF-κB. Metabolism 61 (8), 1142–1151. 10.1016/j.metabol.2012.01.012 22398021

[B78] Lee S. Choi J. Mohanty J. Sousa L. P. Tome F. Pardon E. (2018). Structures of beta-klotho reveal a 'zip code'-like mechanism for endocrine FGF signalling. Nature 553 (7689), 501–505. 10.1038/nature25010 29342135 PMC6594174

[B79] Li X. (2019). The FGF metabolic axis. Front. Med. 13 (5), 511–530. 10.1007/s11684-019-0711-y 31495905 PMC7102389

[B80] Liang Q. Zhong L. Zhang J. Wang Y. Bornstein S. R. Triggle C. R. (2014). FGF21 maintains glucose homeostasis by mediating the cross talk between liver and brain during prolonged fasting. Diabetes 63 (12), 4064–4075. 10.2337/db14-0541 25024372

[B81] Lin Z. Tian H. Lam K. S. L. Lin S. Hoo R. C. L. Konishi M. (2013). Adiponectin mediates the metabolic effects of FGF21 on glucose homeostasis and insulin sensitivity in mice. Cell Metab. 17 (5), 779–789. 10.1016/j.cmet.2013.04.005 23663741

[B82] Liu C. Schonke M. Zhou E. Li Z. Kooijman S. Boon M. R. (2022). Pharmacological treatment with FGF21 strongly improves plasma cholesterol metabolism to reduce atherosclerosis. Cardiovasc. Res. 118 (2), 489–502. 10.1093/cvr/cvab076 33693480 PMC8803070

[B83] Liu X. Zhang P. Martin R. C. Cui G. Wang G. Tan Y. (2016). Lack of fibroblast growth factor 21 accelerates metabolic liver injury characterized by steatohepatities in mice. Am. J. Cancer Res. 6 (5), 1011–1025. 27293995 PMC4889716

[B84] Lundasen T. Hunt M. C. Nilsson L. M. Sanyal S. Angelin B. Alexson S. E. (2007). PPARalpha is a key regulator of hepatic FGF21. Biochem. Biophys. Res. Commun. 360 (2), 437–440. 10.1016/j.bbrc.2007.06.068 17601491

[B85] Lundsgaard A. M. Fritzen A. M. Sjoberg K. A. Myrmel L. S. Madsen L. Wojtaszewski J. (2017). Circulating FGF21 in humans is potently induced by short term overfeeding of carbohydrates. Mol. Metab. 6 (1), 22–29. 10.1016/j.molmet.2016.11.001 28123934 PMC5220397

[B86] Luo Y. Decato B. E. Charles E. D. Shevell D. E. Mcnaney C. Shipkova P. (2022). Pegbelfermin selectively reduces secondary bile acid concentrations in patients with non-alcoholic steatohepatitis. JHEP Rep. 4 (1), 100392. 10.1016/j.jhepr.2021.100392 34977519 PMC8689226

[B87] Makela J. Tselykh T. V. Maiorana F. Eriksson O. Do H. T. Mudo G. (2014). Fibroblast growth factor-21 enhances mitochondrial functions and increases the activity of PGC-1α in human dopaminergic neurons via Sirtuin-1. Springerplus 3, 2. 10.1186/2193-1801-3-2 25932355 PMC4409609

[B88] Markan K. R. Naber M. C. Ameka M. K. Anderegg M. D. Mangelsdorf D. J. Kliewer S. A. (2014). Circulating FGF21 is liver derived and enhances glucose uptake during refeeding and overfeeding. Diabetes 63 (12), 4057–4063. 10.2337/db14-0595 25008183 PMC4238010

[B89] Markan K. R. Potthoff M. J. (2016). Metabolic fibroblast growth factors (FGFs): Mediators of energy homeostasis. Semin. Cell Dev. Biol. 53, 85–93. 10.1016/j.semcdb.2015.09.021 26428296 PMC4814376

[B90] Mashili F. L. Austin R. L. Deshmukh A. S. Fritz T. Caidahl K. Bergdahl K. (2011). Direct effects of FGF21 on glucose uptake in human skeletal muscle: Implications for type 2 diabetes and obesity. Diabetes Metab. Res. Rev. 27 (3), 286–297. 10.1002/dmrr.1177 21309058

[B91] Monte M. J. Marin J. J. Antelo A. Vazquez-Tato J. (2009). Bile acids: Chemistry, physiology, and pathophysiology. World J. Gastroenterol. 15 (7), 804–816. 10.3748/wjg.15.804 19230041 PMC2653380

[B92] Mudaliar S. Henry R. R. Sanyal A. J. Morrow L. Marschall H. U. Kipnes M. (2013). Efficacy and safety of the farnesoid X receptor agonist obeticholic acid in patients with type 2 diabetes and nonalcoholic fatty liver disease. Gastroenterology 145 (3), 574–582. e1. 10.1053/j.gastro.2013.05.042 23727264

[B93] Muller T. D. Tschop M. H. (2014). Play down protein to play up metabolism? J. Clin. investigation 124 (9), 3691–3693. 10.1172/JCI77508 PMC4151229 25133420

[B94] Nishimura T. Nakatake Y. Konishi M. Itoh N. (2000). Identification of a novel FGF, FGF-21, preferentially expressed in the liver. Biochim. Biophys. Acta 1492 (1), 203–206. 10.1016/s0167-4781(00)00067-1 10858549

[B95] Ogawa Y. Kurosu H. Yamamoto M. Nandi A. Rosenblatt K. P. Goetz R. (2007). BetaKlotho is required for metabolic activity of fibroblast growth factor 21. Proc. Natl. Acad. Sci. U. S. A. 104 (18), 7432–7437. 10.1073/pnas.0701600104 17452648 PMC1855074

[B96] Oishi K. Konishi M. Murata Y. Itoh N. (2011). Time-imposed daily restricted feeding induces rhythmic expression of Fgf21 in white adipose tissue of mice. Biochem. Biophys. Res. Commun. 412 (2), 396–400. 10.1016/j.bbrc.2011.07.125 21835167

[B97] Orava J. Nuutila P. Lidell M. E. Oikonen V. Noponen T. Viljanen T. (2011). Different metabolic responses of human Brown adipose tissue to activation by cold and insulin. Cell Metab. 14 (2), 272–279. 10.1016/j.cmet.2011.06.012 21803297

[B98] Owen B. M. Bookout A. L. Ding X. Lin V. Y. Atkin S. D. Gautron L. (2013). FGF21 contributes to neuroendocrine control of female reproduction. Nat. Med. 19 (9), 1153–1156. 10.1038/nm.3250 23933983 PMC3769455

[B99] Pan Y. Hui X. Hoo R. Ye D. Chan C. Feng T. (2019). Adipocyte-secreted exosomal microRNA-34a inhibits M2 macrophage polarization to promote obesity-induced adipose inflammation. J. Clin. Invest. 129 (2), 834–849. 10.1172/JCI123069 30667374 PMC6355214

[B100] Patel V. Adya R. Chen J. Ramanjaneya M. Bari M. F. Bhudia S. K. (2014). Novel insights into the cardio-protective effects of FGF21 in lean and obese rat hearts. PLoS One 9 (2), e87102. 10.1371/journal.pone.0087102 24498293 PMC3911936

[B101] Planavila A. Redondo I. Hondares E. Vinciguerra M. Munts C. Iglesias R. (2013). Fibroblast growth factor 21 protects against cardiac hypertrophy in mice. Nat. Commun. 4, 2019. 10.1038/ncomms3019 23771152

[B102] Planavila A. Redondo-Angulo I. Ribas F. Garrabou G. Casademont J. Giralt M. (2015). Fibroblast growth factor 21 protects the heart from oxidative stress. Cardiovasc. Res. 106 (1), 19–31. 10.1093/cvr/cvu263 25538153

[B103] Potthoff M. J. Inagaki T. Satapati S. Ding X. He T. Goetz R. (2009). FGF21 induces PGC-1alpha and regulates carbohydrate and fatty acid metabolism during the adaptive starvation response. Proc. Natl. Acad. Sci. U. S. A. 106 (26), 10853–10858. 10.1073/pnas.0904187106 19541642 PMC2705613

[B104] Rosales-Soto G. Diaz-Vegas A. Casas M. Contreras-Ferrat A. Jaimovich E. (2020). Fibroblast growth factor-21 potentiates glucose transport in skeletal muscle fibers. J. Mol. Endocrinol. 65 (3), 85–95. 10.1530/JME-19-0210 32755998

[B105] Rosen E. D. Spiegelman B. M. (2014). What we talk about when we talk about fat. Cell 156 (1-2), 20–44. 10.1016/j.cell.2013.12.012 24439368 PMC3934003

[B106] Samms R. J. Smith D. P. Cheng C. C. Antonellis P. P. Perfield J. N. Kharitonenkov A. (2015). Discrete aspects of FGF21 *in vivo* Pharmacology do not require UCP1. Cell Rep. 11 (7), 991–999. 10.1016/j.celrep.2015.04.046 25956583

[B107] Sanyal A. Charles E. D. Neuschwander-Tetri B. A. Loomba R. Harrison S. A. Abdelmalek M. F. (2019). Pegbelfermin (BMS-986036), a PEGylated fibroblast growth factor 21 analogue, in patients with non-alcoholic steatohepatitis: A randomised, double-blind, placebo-controlled, phase 2a trial. Lancet 392 (10165), 2705–2717. 10.1016/S0140-6736(18)31785-9 30554783

[B108] Schlein C. Talukdar S. Heine M. Fischer A. W. Krott L. M. Nilsson S. K. (2016). FGF21 lowers plasma triglycerides by accelerating lipoprotein catabolism in white and Brown adipose tissues. Cell Metab. 23 (3), 441–453. 10.1016/j.cmet.2016.01.006 26853749

[B109] Schreuder T. C. Marsman H. A. Lenicek M. van Werven J. R. Nederveen A. J. Jansen P. L. (2010). The hepatic response to FGF19 is impaired in patients with nonalcoholic fatty liver disease and insulin resistance. Am. J. Physiol. Gastrointest. Liver Physiol. 298 (3), G440–G445. 10.1152/ajpgi.00322.2009 20093562

[B110] Singhal G. Douris N. Fish A. J. Zhang X. Adams A. C. Flier J. S. (2016a). Fibroblast growth factor 21 has no direct role in regulating fertility in female mice. Mol. Metab. 5 (8), 690–698. 10.1016/j.molmet.2016.05.010 27656406 PMC5021666

[B111] Singhal G. Fisher F. M. Chee M. J. Tan T. G. El O. A. Adams A. C. (2016b). Fibroblast growth factor 21 (FGF21) protects against high fat diet induced inflammation and islet hyperplasia in pancreas. PLoS One 11 (2), e0148252. 10.1371/journal.pone.0148252 26872145 PMC4752212

[B112] Smith R. Duguay A. Bakker A. Li P. Weiszmann J. Thomas M. R. (2013). FGF21 can be mimicked *in vitro* and *in vivo* by a novel anti-FGFR1c/β-Klotho bispecific protein. PLoS One 8 (4), e61432. 10.1371/journal.pone.0061432 23630589 PMC3632592

[B113] So W. Y. Cheng Q. Chen L. Evans-Molina C. Xu A. Lam K. S. (2013). High glucose represses beta-klotho expression and impairs fibroblast growth factor 21 action in mouse pancreatic islets: Involvement of peroxisome proliferator-activated receptor gamma signaling. Diabetes 62 (11), 3751–3759. 10.2337/db13-0645 23897951 PMC3806592

[B114] Spann R. A. Morrison C. D. den Hartigh L. J. (2021). The nuanced metabolic functions of endogenous FGF21 depend on the nature of the stimulus, tissue source, and experimental model. Front. Endocrinol. (Lausanne) 12, 802541. 10.3389/fendo.2021.802541 35046901 PMC8761941

[B115] Stanislaus S. Hecht R. Yie J. Hager T. Hall M. Spahr C. (2017). A novel fc-FGF21 with improved resistance to proteolysis, increased affinity toward beta-klotho, and enhanced efficacy in mice and cynomolgus monkeys. Endocrinology 158 (5), 1314–1327. 10.1210/en.2016-1917 28324011

[B116] Sui Y. Chen J. (2022). Hepatic FGF21: Its emerging role in inter-organ crosstalk and cancers. Int. J. Biol. Sci. 18 (15), 5928–5942. 10.7150/ijbs.76924 36263162 PMC9576513

[B117] Sunaga H. Koitabashi N. Iso T. Matsui H. Obokata M. Kawakami R. (2019). Activation of cardiac AMPK-FGF21 feed-forward loop in acute myocardial infarction: Role of adrenergic overdrive and lipolysis byproducts. Sci. Rep. 9 (1), 11841. 10.1038/s41598-019-48356-1 31413360 PMC6694166

[B118] Suzuki M. Uehara Y. Motomura-Matsuzaka K. Oki J. Koyama Y. Kimura M. (2008). betaKlotho is required for fibroblast growth factor (FGF) 21 signaling through FGF receptor (FGFR) 1c and FGFR3c. Mol. Endocrinol. 22 (4), 1006–1014. 10.1210/me.2007-0313 18187602 PMC5419549

[B119] Szczepańska E. Gietka-Czernel M. (2022). FGF21: A novel regulator of glucose and lipid metabolism and whole-body energy balance. Horm. Metab. Res. 54 (4), 203–211. 10.1055/a-1778-4159 35413740

[B120] Talukdar S. Owen B. M. Song P. Hernandez G. Zhang Y. Zhou Y. (2016a). FGF21 regulates sweet and alcohol preference. Cell Metab. 23 (2), 344–349. 10.1016/j.cmet.2015.12.008 26724861 PMC4749404

[B121] Talukdar S. Zhou Y. Li D. Rossulek M. Dong J. Somayaji V. (2016b). A long-acting FGF21 molecule, PF-05231023, decreases body weight and improves lipid profile in non-human primates and type 2 diabetic subjects. Cell Metab. 23 (3), 427–440. 10.1016/j.cmet.2016.02.001 26959184

[B122] Tanaka N. Takahashi S. Zhang Y. Krausz K. W. Smith P. B. Patterson A. D. (2015). Role of fibroblast growth factor 21 in the early stage of NASH induced by methionine- and choline-deficient diet. Biochim. Biophys. Acta 1852 (7), 1242–1252. 10.1016/j.bbadis.2015.02.012 25736301 PMC4433820

[B123] Tang T. T. Li Y. Y. Li J. J. Wang K. Han Y. Dong W. Y. (2018). Liver-heart crosstalk controls IL-22 activity in cardiac protection after myocardial infarction. Theranostics 8 (16), 4552–4562. 10.7150/thno.24723 30214638 PMC6134935

[B124] Tanimura Y. Aoi W. Takanami Y. Kawai Y. Mizushima K. Naito Y. (2016). Acute exercise increases fibroblast growth factor 21 in metabolic organs and circulation. Physiol. Rep. 4 (12), e12828. 10.14814/phy2.12828 27335433 PMC4923231

[B125] Tezze C. Romanello V. Sandri M. (2019). FGF21 as modulator of metabolism in health and disease. Front. Physiol. 10, 419. 10.3389/fphys.2019.00419 31057418 PMC6478891

[B126] Thompson W. C. Zhou Y. Talukdar S. Musante C. J. (2016). PF-05231023, a long-acting FGF21 analogue, decreases body weight by reduction of food intake in non-human primates. J. Pharmacokinet. Phar. 43 (4), 411–425. 10.1007/s10928-016-9481-1 PMC4954843 27405817

[B127] Tucker W. Tucker B. Rye K. Ong K. L. (2022). Fibroblast growth factor 21 in heart failure. Heart fail. Rev. 10.1007/s10741-022-10268-0 PMC9902422 36028609

[B128] Uonaga T. Toyoda K. Okitsu T. Zhuang X. Yamane S. Uemoto S. (2010). FGF-21 enhances islet engraftment in mouse syngeneic islet transplantation model. Islets 2 (4), 247–251. 10.4161/isl.2.4.12402 21099319 PMC3322536

[B129] Vernia S. Lee A. Kennedy N. J. Han M. S. Isasa M. Cavanagh-Kyros J. (2022). Phosphorylation of RXRα mediates the effect of JNK to suppress hepatic FGF21 expression and promote metabolic syndrome. Proc. Natl. Acad. Sci. U. S. A. 119 (44), e2210434119. 10.1073/pnas.2210434119 36282921 PMC9636906

[B130] von Holstein-Rathlou S. Bondurant L. D. Peltekian L. Naber M. C. Yin T. C. Claflin K. E. (2016). FGF21 mediates endocrine control of simple sugar intake and sweet taste preference by the liver. Cell Metab. 23 (2), 335–343. 10.1016/j.cmet.2015.12.003 26724858 PMC4756759

[B131] Wang Q. Yuan J. Yu Z. Lin L. Jiang Y. Cao Z. (2018). FGF21 attenuates high-fat diet-induced cognitive impairment via metabolic regulation and anti-inflammation of obese mice. Mol. Neurobiol. 55 (6), 4702–4717. 10.1007/s12035-017-0663-7 28712011 PMC5971086

[B132] Wei W. Dutchak P. A. Wang X. Ding X. Wang X. Bookout A. L. (2012). Fibroblast growth factor 21 promotes bone loss by potentiating the effects of peroxisome proliferator-activated receptor γ. Proc. Natl. Acad. Sci. U. S. A. 109 (8), 3143–3148. 10.1073/pnas.1200797109 22315431 PMC3286969

[B133] Weng Y. Chabot J. R. Bernardo B. Yan Q. Zhu Y. Brenner M. B. (2015). Pharmacokinetics (PK), pharmacodynamics (PD) and integrated PK/PD modeling of a novel long acting FGF21 clinical candidate PF-05231023 in diet-induced obese and leptin-deficient obese mice. PLoS One 10 (3), e0119104. 10.1371/journal.pone.0119104 25790234 PMC4366384

[B134] Wente W. Efanov A. M. Brenner M. Kharitonenkov A. Koster A. Sandusky G. E. (2006). Fibroblast growth factor-21 improves pancreatic beta-cell function and survival by activation of extracellular signal-regulated kinase 1/2 and Akt signaling pathways. Diabetes 55 (9), 2470–2478. 10.2337/db05-1435 16936195

[B135] Wong C. Dash A. Fredrickson J. Lewin-Koh N. Chen S. Yoshida K. (2022). Fibroblast growth factor receptor 1/klothoβ agonist BFKB8488A improves lipids and liver health markers in patients with diabetes or NAFLD: A phase 1b randomized trial. Hepatology. 10.1002/hep.32742 35993161

[B136] Xu J. Stanislaus S. Chinookoswong N. Lau Y. Y. Hager T. Patel J. (2009). Acute glucose-lowering and insulin-sensitizing action of FGF21 in insulin-resistant mouse models--association with liver and adipose tissue effects. Am. J. Physiol. Endocrinol. Metab. 297 (5), E1105–E1114. 10.1152/ajpendo.00348.2009 19706786

[B137] Yang C. Jin C. Li X. Wang F. Mckeehan W. L. Luo Y. (2012). Differential specificity of endocrine FGF19 and FGF21 to FGFR1 and FGFR4 in complex with KLB. PLoS One 7 (3), e33870. 10.1371/journal.pone.0033870 22442730 PMC3307775

[B138] Yang W. Liu L. Wei Y. Fang C. Zhou F. Chen J. (2019). Exercise ameliorates the FGF21-adiponectin axis impairment in diet-induced obese mice. Endocr. Connect. 8 (5), 596–604. 10.1530/EC-19-0034 30978696 PMC6510890

[B139] Yao Q. Y. Xu B. L. Wang J. Y. Liu H. C. Zhang S. C. Tu C. T. (2012). Inhibition by curcumin of multiple sites of the transforming growth factor-beta1 signalling pathway ameliorates the progression of liver fibrosis induced by carbon tetrachloride in rats. BMC Complement. Altern. Med. 12, 156. 10.1186/1472-6882-12-156 22978413 PMC3495222

[B140] Ye D. Wang Y. Li H. Jia W. Man K. Lo C. M. (2014). Fibroblast growth factor 21 protects against acetaminophen-induced hepatotoxicity by potentiating peroxisome proliferator-activated receptor coactivator protein-1α-mediated antioxidant capacity in mice. Hepatology 60 (3), 977–989. 10.1002/hep.27060 24590984

[B141] Yie J. Wang W. Deng L. Tam L. T. Stevens J. Chen M. M. (2012). Understanding the physical interactions in the FGF21/FGFR/β-Klotho complex: Structural requirements and implications in FGF21 signaling. Chem. Biol. Drug Des. 79 (4), 398–410. 10.1111/j.1747-0285.2012.01325.x 22248288

[B142] Yin J. Bao L. Chen R. Gao W. Gao X. Yao W. (2018). Enhanced expression and distinctive characterization of a long-acting FGF21 and its potential to alleviate nonalcoholic steatohepatitis. Biochimie 151, 166–175. 10.1016/j.biochi.2018.05.020 29870802

[B143] Younossi Z. Tacke F. Arrese M. Chander S. B. Mostafa I. Bugianesi E. (2019). Global perspectives on nonalcoholic fatty liver disease and nonalcoholic steatohepatitis. Hepatology 69 (6), 2672–2682. 10.1002/hep.30251 30179269

[B144] Yu Y. Bai F. Liu Y. Yang Y. Yuan Q. Zou D. (2015). Fibroblast growth factor (FGF21) protects mouse liver against D-galactose-induced oxidative stress and apoptosis via activating Nrf2 and PI3K/Akt pathways. Mol. Cell. Biochem. 403 (1-2), 287–299. 10.1007/s11010-015-2358-6 25701356

[B145] Zakharia K. Tabibian A. Lindor K. D. Tabibian J. H. (2018). Complications, symptoms, quality of life and pregnancy in cholestatic liver disease. Liver Int. 38 (3), 399–411. 10.1111/liv.13591 28921801

[B146] Zarei M. Barroso E. Palomer X. Dai J. Rada P. Quesada-Lopez T. (2018). Hepatic regulation of VLDL receptor by PPARβ/δ and FGF21 modulates non-alcoholic fatty liver disease. Mol. Metab. 8, 117–131. 10.1016/j.molmet.2017.12.008 29289645 PMC5985050

[B147] Zhang X. Ibrahimi O. A. Olsen S. K. Umemori H. Mohammadi M. Ornitz D. M. (2006). Receptor specificity of the fibroblast growth factor family. The complete mammalian FGF family. J. Biol. Chem. 281 (23), 15694–15700. 10.1074/jbc.M601252200 16597617 PMC2080618

[B148] Zhang Y. Liu D. Long X. Fang Q. Jia W. Li H. (2021). The role of FGF21 in the pathogenesis of cardiovascular disease. Chin. Med. J.-Peking. 134 (24), 2931–2943. 10.1097/CM9.0000000000001890 PMC8710326 34939977

